# Panoramic snapshot of serum soluble mediator interplay in pregnant women with convalescent COVID-19: an exploratory study

**DOI:** 10.3389/fimmu.2023.1176898

**Published:** 2023-04-12

**Authors:** Geraldo Magela Fernandes, Lizandra Moura Paravidine Sasaki, Gabriela Profírio Jardim-Santos, Heidi Luise Schulte, Felipe Motta, Ângelo Pereira da Silva, Aleida Oliveira de Carvalho, Yacara Ribeiro Pereira, Caroline de Oliveira Alves, David Alves de Araújo Júnior, Dayde Lane Mendonça-Silva, Karina Nascimento Costa, Maria Eduarda Canellas de Castro, Lucas Lauand, Rodrigo de Resende Nery, Rosana Tristão, Patricia Shu Kurizky, Otávio de Toledo Nóbrega, Laila Salmen Espindola, Luiz Cláudio Gonçalves de Castro, Patrícia Nessralla Alpoim, Lara Carvalho Godoi, Luci Maria Sant Ana Dusse, Jordana Grazziela Alves Coelho-dos-Reis, Laurence Rodrigues do Amaral, Matheus de Souza Gomes, Pedro Luiz Lima Bertarini, Joaquim Pedro Brito-de-Sousa, Ismael Artur da Costa-Rocha, Ana Carolina Campi-Azevedo, Vanessa Peruhype-Magalhães, Andrea Teixeira-Carvalho, Alberto Moreno Zaconeta, Alexandre Anderson de Sousa Munhoz Soares, Valéria Valim, Ciro Martins Gomes, Cleandro Pires de Albuquerque, Olindo Assis Martins-Filho, Licia Maria Henrique da Mota

**Affiliations:** ^1^ Programa de Pós-Graduação em Ciências Médicas, Universidade de Brasília (UnB), Brasília, Brazil; ^2^ Hospital Universitário de Brasília, Universidade de Brasília (UnB), Brasília, Brazil; ^3^ Faculdade de Medicina, Universidade de Brasília (UnB), Brasília, Brazil; ^4^ Faculdade de Farmácia, Universidade Federal de Minas Gerais, Belo Horizonte, Brazil; ^5^ Laboratório de Virologia Básica e Aplicada, Instituto de Ciências Biológicas, Universidade Federal de Minas Gerais, Belo Horizonte, Brazil; ^6^ Laboratório de Bioinformática e Análises Moleculares, Universidade Federal de Uberlândia, Patos de Minas, Brazil; ^7^ Instituto René Rachou, Fundação Oswaldo Cruz (FIOCRUZ-Minas), Belo Horizonte, Brazil; ^8^ Hospital Universitário Cassiano Antônio Moraes, Universidade Federal do Espírito Santo (HUCAM-UFES), Vitória, Brazil; ^9^ Programa de Pós-Graduação em Saúde Coletiva (PPGSC), Centro de Ciências Médicas, Universidade Federal do Espírito Santo, Vitória, Brazil; ^10^ Programa de Pós-Graduação em Patologia Molecular, Universidade de Brasília (UnB), Brasília, Brazil

**Keywords:** chemokines, cytokines, growth factors, COVID-19, pregnancy

## Abstract

**Introduction:**

SARS-CoV-2 infection during pregnancy can induce changes in the maternal immune response, with effects on pregnancy outcome and offspring. This is a cross-sectional observational study designed to characterize the immunological status of pregnant women with convalescent COVID-19 at distinct pregnancy trimesters. The study focused on providing a clear snapshot of the interplay among serum soluble mediators.

**Methods:**

A sample of 141 pregnant women from all prenatal periods (1^st^, 2^nd^ and 3^rd^ trimesters) comprised patients with convalescent SARS-CoV-2 infection at 3-20 weeks after symptoms onset (COVID, n=89) and a control group of pre-pandemic non-infected pregnant women (HC, n=52). Chemokine, pro-inflammatory/regulatory cytokine and growth factor levels were quantified by a high-throughput microbeads array.

**Results:**

In the HC group, most serum soluble mediators progressively decreased towards the 2^nd^ and 3^rd^ trimesters of pregnancy, while higher chemokine, cytokine and growth factor levels were observed in the COVID patient group. Serum soluble mediator signatures and heatmap analysis pointed out that the major increase observed in the COVID group related to pro-inflammatory cytokines (IL-6, TNF-α, IL-12, IFN-γ and IL-17). A larger set of biomarkers displayed an increased COVID/HC ratio towards the 2^nd^ (3x increase) and the 3^rd^ (3x to 15x increase) trimesters. Integrative network analysis demonstrated that HC pregnancy evolves with decreasing connectivity between pairs of serum soluble mediators towards the 3^rd^ trimester. Although the COVID group exhibited a similar profile, the number of connections was remarkably lower throughout the pregnancy. Meanwhile, IL-1Ra, IL-10 and GM-CSF presented a preserved number of correlations (≥5 strong correlations in HC and COVID), IL-17, FGF-basic and VEGF lost connectivity throughout the pregnancy. IL-6 and CXCL8 were included in a set of acquired attributes, named COVID-selective (≥5 strong correlations in COVID and <5 in HC) observed at the 3^rd^ pregnancy trimester.

**Discussion and conclusion:**

From an overall perspective, a pronounced increase in serum levels of soluble mediators with decreased network interplay between them demonstrated an imbalanced immune response in convalescent COVID-19 infection during pregnancy that may contribute to the management of, or indeed recovery from, late complications in the post-symptomatic phase of the SARS-CoV-2 infection in pregnant women.

## 1 Introduction

In March 2020, the World Health Organization (WHO) characterized the outbreak of the Severe Acute Respiratory Syndrome Coronavirus 2 (SARS-CoV-2) disease (COVID-19) as a pandemic, having since confirmed more than 655 million COVID-19 cases, 6.6 million of which resulted in death (1). SARS-CoV-2 is transmitted through airborne droplets, respiratory secretions, and direct contact. The clinical symptoms relating to the COVID-19 disease were primarily respiratory, and later reported as multisystemic effects. COVID-19 illness symptoms can be asymptomatic, mild, moderate, severe, or critical (2–4). Fever, cough, dyspnea, and myalgia were the most common mild symptoms. The pathogenesis of COVID-19 has been strongly associated with an unbalanced immune response; however, the pathophysiology of the disease remains under investigation (5–7).

Multiple studies concluded that pregnant women are a high-risk population for the COVID-19 disease. Infectious diseases in pregnancy are regularly considered a critical condition. Physiological changes during pregnancy have significant effects on the immune system, cardiopulmonary system and coagulation, and these changes may result in an altered response to COVID-19 infection (3, 8–11). Cytokine levels during pregnancy could be responsible for metabolic imprinting as cytokines are transferable from maternal to fetal circulation and are capable of modulating placental nutrient transfer. Maternal inflammation may induce metabolic reprogramming at several levels, from the periconceptional period onwards. Such processes and their consequences on the maternal and perinatal periods have not been extensively studied to date. Moreover, the maternal immune activation triggered by COVID-19 can have impacts for the mother, pregnancy outcome and offspring (12, 13). The understanding such phenomena should contribute to the proper management of children born to SARS-CoV-2-infected mothers (14).

The aim of the present study was to conduct a prospective observational study designed to characterize the immunological status of pregnant women with convalescent COVID-19, focusing on an overall snapshot of the interplay between serum soluble mediators.

## 2 Materials and methods

### 2.1 Study population

This cross-sectional observational study was conducted between July 2020 and December 2021 during the COVID-19 pandemic in the Federal District of Brazil during circulation of the SARS-CoV-2 B.1.1.28 and B.1.1.33 strains. A total of 141 participants were enrolled as non-probability convenience sampling, including pregnant women with convalescent SARS-CoV-2 infection (COVID, n=89) at 3-20 weeks after symptoms onset during the prenatal period (1^st^, 2^nd^ and 3^rd^ trimesters), together with a healthy control group composed of age-matched pre-pandemic non-infected pregnant women (HC, n=52).

The COVID-19 pregnant women were recruited at two public hospitals - the University Hospital of Brasília and the Asa Norte Regional Hospital, both public reference centers for COVID-19 in the Federal District of Brazil and participants of a large research project named PROUDEST (15). The COVID group comprised pregnant women aged 18-44 years, with a median age of 31 years. This group was further categorized into subgroups according to the pregnancy trimester, referred to as: 1^st^ (n=7), 2^nd^ (n=34) and 3^rd^ (n=48). COVID-19 diagnosis was confirmed by a documented positive RT-PCR test using a nasopharyngeal swab or rapid test (Biomanguinhos, FIOCRUZ, Brazil) for IgM or IgG, during pregnancy. Most of the COVID-19 group (97%, 86 out of 89) presented the non-severe form of the disease. The most common symptoms were: Anosmia (68%), runny nose and/or nasal congestion (68%), headache (67%), ageusia (63%), myalgia (57%), cough (43%), fever (43%), dyspnea (31%), sore throat (31%), asthenia (22%), diarrhea (17%), nausea and vomiting (11%), joint pain (5%), dizziness (4%) and skin diseases (2%). SARS-CoV-2 infection during pregnancy was associated with important adverse maternal and neonatal outcomes, including gestational diabetes mellitus (37%), Apgar score at first minute ≤ 7 (22%), systemic arterial hypertension (18%), fetal restriction growth (11%), preterm labor (11%), acute fetal distress (8%), Apgar score at fifth minute ≤ 7 (5%) and preeclampsia (3%).

The HC group comprised a selected non-probability convenience sampling from a biorepository maintained at Grupo Integrado de Pesquisas em Biomarcadores, Instituto René Rachou, Fundação Oswaldo Cruz (FIOCRUZ-Minas), Belo Horizonte, Brazil. The HC group comprised pregnant women, aged 18- 42 years, with a median age of 28 years. The healthy control group was composed by primiparous with no previous history or current status of obesity, systemic arterial hypertension, diabetes mellitus and without records of pre-eclampsia. The HC group was further categorized into subgroups according to pregnancy trimester, referred to as: 1^st^ (n=21), 2^nd^ (n=10) and 3^rd^ (n=21).

The **Table 1** summarize the major demographic and clinical features of the study population.

**Table 1:** Demographic and clinical features of the study population.

Characteristics	GROUPS	
	Healthy Controls – HC (n=52)	COVID-19 – COVID (n=89)
**Age, median (min-max)**	28 (18-42)	31 (18-44)
Obstetric History		
Previous Pregnancies, median (min-max)	0% (0)	2 (0-6)
Abortions, median (min-max)	0% (0)	0 (0-2)
Complications* % (n)	0% (0)	24% (21)
Current Study		
Obesity % (n)	0% (0)	11% (10)
SAH % (n)	0% (0)	18% (16)
Diabetes % (n)	0% (0)	37% (33)
Pre-eclampsia % (n)	0% (0)	3% (3)

SAH, systemic arterial hypertension. *Obesity, SAH, diabetes, pre-eclampsia.

All study participants provided written informed consent prior to inclusion in accordance with the Helsinki Declaration and Resolution 466/2012 from the Brazilian National Health Council for research involving human subjects. This study was recorded on the Brazilian Registry of Clinical Trials Platform (ReBEC, RBR-65qxs2) and approved by the National Commission for Ethics in Research in Brazil (CONEP, CAAE 32359620.0.0000.5558). The anonymization strategy to protect the identity of participants was achieved by replacing the direct identifiers by standardized alphanumeric codes (*PRAxxxPNy and PRBxxxPNy*), where “*PR*” refer to the PROUDEST project name (15), “*A*” and “*B*” refers to the hospital unit, the “*xxx*” represent the sequential number of patient inclusion, “*PN*” refer to prenatal period and “*y*” the trimester of sample collection.

### 2.2 Biological samples

Whole blood sample (10 mL) were collected from each participant in vacuum tubes without anticoagulant by venipuncture at the first prenatal appointment or upon enrolment in the study. Serum samples were obtained by centrifugation (1400 x g, 10 min, 4°C) of original samples within 6 h after blood collection. The serum specimens were aliquoted and stored at -80°C until quantification of serum soluble mediators.

### 2.3 Quantification of serum soluble mediators

Serum soluble mediators were quantified by a high-throughput Luminex microbead multiplex assay (Bio-Plex Pro™ Human Cytokine 27-plex Assay, Bio-Rad Laboratories, Hercules, CA, USA). The manufacturer’s instructions were followed to determine the concentrations of chemokines (CXCL8; CCL11; CCL3; CCL4; CCL2; CCL5; CXCL10), pro-inflammatory cytokines (IL-1β; IL-6; TNF-α; IL-12; IFN-γ; IL-15; IL-17), regulatory cytokines (IL-1Ra; IL-4; IL-5; IL-9; IL-10; IL-13) and growth factors (FGF-basic; VEGF; PDGF; G-CSF; GM-CSF; IL-2; IL-7). The assays were conducted in parallel batches by a trained technician at the flow cytometry facility at FIOCRUZ-Minas. The concentrations of serum soluble mediators (pg/mL) were obtained according to a 5-parameter logistic curve fit regression of standard curves.

### 2.4 Statistical analysis

Descriptive statistics were carried out using the Prism 8.0.2 software (GraphPad Software, San Diego, USA). Data normality was assessed using the Shapiro-Wilk test. Considering the nonparametric distribution of all data sets, multiple comparisons amongst HC and COVID subgroups were carried out using the Kruskal-Wallis followed by Dunn’s post-test. Additionally, comparative analysis between HC and COVID at matching trimesters was performed using the Mann-Whitney test. In all cases, statistical significance was considered at p<0.05.

The serum soluble mediator signatures were calculated as the proportion (%) of pregnant women with serum levels above the reference values (cut-off) defined as the median Z-score of each soluble mediator detected for all HC along the 1^st^, 2^nd^ and 3^rd^ trimesters (CXCL8=-0.3; CCL11=-0.3; CCL3=-0.3; CCL4=-0.3; CCL2=-0.4; CCL5=-0.2; CXCL10=-0.2; IL-1β=-0.3; IL-6=-0.3; TNF-α=-0.3; IL-12=-0.3; IFN-γ=-0.4; IL-15=-0.5; IL-17=-0.4; IL-1Ra=-0.4; IL-4=-0.3; IL-5=-0.2; IL-9=-0.2; IL-10=-0.2; IL-13=-0.4; FGF-basic=-0.5; PDGF=-0.4; VEGF=-0.4; G-CSF=-0.2; GM-CSF=-0.4; IL-2=-0.4; IL-7=-0.3). Additionally, trimester-matching signatures were assembled, considering the reference values (cut-off) defined as the median Z-score of each soluble mediator detected for HC trimester subgroups at 1^st^ (CXCL8 = 0.2; CCL11 = 0.1; CCL3 = 0; CCL4=-0.6; CCL2 = 0.4; CCL5=-0.2; CXCL10=-0.7; IL-1β=-0.2; IL-6 = 1.6; TNF-α=-0.1; IL-12=-0.2; IFN-γ=0.1; IL-15=-0.5; IL-17 = 0.1; IL-1Ra=0.1; IL-4=-0.5; IL-5=-0.1; IL-9 = 0.5; IL-10=-0.2; IL-13 = 0.2; FGF-basic=0.3; PDGF=0.2; VEGF=0.1; G-CSF=-0.2; GM-CSF=0.1; IL-2=-0.4; IL-7=-0.2), 2^nd^ (CXCL8=-0.3; CCL11=-0.2; CCL3=-0.3; CCL4=-0.2; CCL2=-0.5; CCL5=-0.1; CXCL10=-0.6; IL-1β=-0.3; IL-6=-0.4; TNF-α=-0.3; IL-12=-0.3; IFN-γ=-0.4; IL-15 = 0.2; IL-17=-0.2; IL-1Ra=-0.5; IL-4 = 0.2; IL-5=-0.2; IL-9=-0.6; IL-10=-0.2; IL-13=-0.4; FGF-basic=-0.6; PDGF=-0.6; VEGF=-0.2; G-CSF=-0.1; GM-CSF=-0.6; IL-2=-0.1; IL-7=-0.2) and 3^rd^ trimesters (CXCL8=-0.4; CCL11=-0.4; CCL3=-0.3; CCL4 = 0.3; CCL2=-0.5; CCL5 = 0.1; CXCL10 = 0.6; IL-1β=-0.4; IL-6=-0.3; TNF-α=-0.3; IL-12=-0.3; IFN-γ=-0.4; IL-15=-0.5; IL-17=-0.4; IL-1Ra=-0.6; IL-4=-0.3; IL-5=-0.2; IL-9=-0.8; IL-10=-0.3; IL-13=-0.4; FGF-basic=-0.6; PDGF=-0.7; VEGF=-0.4; G-CSF=-0.2; GM-CSF=-0.7; IL-2=-0.5; IL-7=-0.3). The serum soluble mediators displaying a proportion above 50% in pregnant women were included in the set of biomarkers with increased levels.

Heatmap constructs were assembled using conditional formatting in Microsoft Excel to illustrate the overall profile of serum soluble mediator signatures of the COVID and HC subgroups along the pregnancy trimesters. The ratio between the proportion of pregnant women with serum levels above the reference values in the COVID group in relation to HC (%COVID/%HC) was also assessed by comparative analysis.

Serum-soluble mediator networks were built based on correlation analysis (Pearson and Spearman rank tests) between pairs of serum-soluble mediators. Only significant strong correlations (p<0.05 and “r” scores ≥ |0.67|) were employed to construct the comprehensive networks. The open-source Cytoscape software (available at https://cytoscape.org) was used to create cluster network layouts comprising the 4 categories of serum soluble mediators - chemokines, pro-inflammatory cytokines, regulatory cytokines, and growth factors. Descriptive analysis of serum soluble mediator networks was performed by considering the ascendant number of strong correlations to identify the set of biomarkers with five or more strong correlations (≥ 5). Venn Diagram analysis (available at (http://bioinformatics.psb.ugent.be/webtools/Venn/) was performed to assess the preserved (common), lost or acquired (selective) serum soluble mediators with ≥ 5 strong correlations in COVID subgroups compared to trimester-matching HC.

The MATLAB software was employed for Principal Component Analysis (PCA). The PCA data was assembled to verify the ability of serum soluble mediators to cluster convalescent COVID-19 pregnant women from HC, as well as subgroups of COVID-19 as compared to trimester-matching HC. The PCA analysis enabled data dimensionality reduction.

## 3 Results

### 3.1 Levels of serum soluble mediators in convalescent COVID-19 at distinct pregnancy trimesters

**Figure 1:**
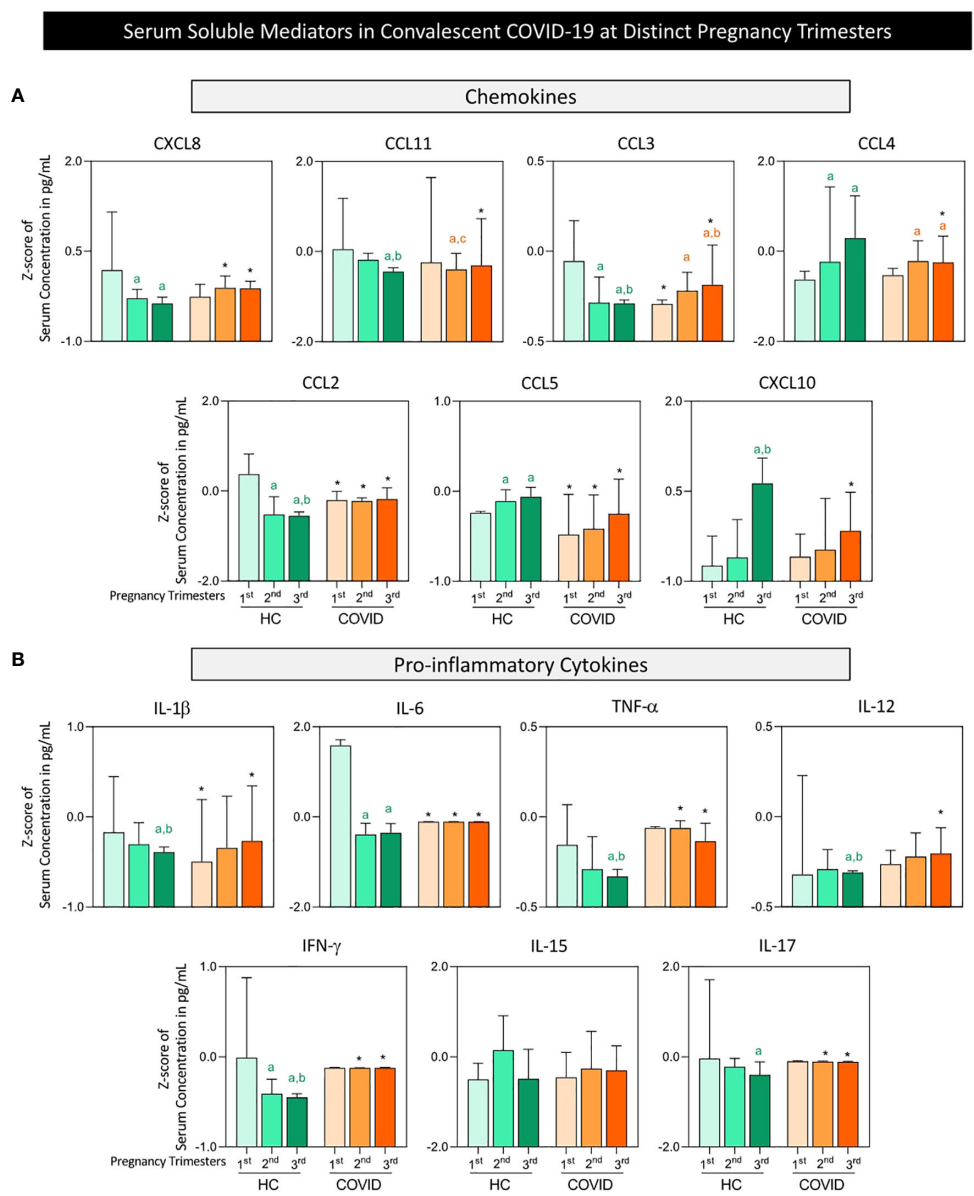
Serum and pro-inflammatory cytokines in convalescent COVID-19 patients at distinct pregnancy trimesters. The levels of: **(A)** chemokines (CXCL8, CCL11, CCL3, CCL4, CCL2, CCL5, CXCL10), and **(B)** pro-inflammatory cytokines (IL-1β, IL-6, TNF-α, IL-12, IFN-γ, IL-15, IL -17) were measured in serum samples from pregnant women with convalescent COVID-19 at 3-20 weeks after symptoms onset (COVID, n=89), with pre-pandemic non-infected pregnant women as a Healthy Control (HC, n=52). The HC and COVID-19 groups were further categorized into subgroups according to pregnancy trimester, referred to as: HC 1^st^ (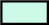, n=21), HC 2^nd^ (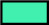, n=10), HC 3^rd^ (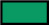, n=21), and COVID 1^st^ (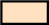, n=7), COVID 2^nd^ (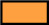, n=34), COVID 3^rd^ (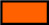, n=48). The measurements were taken by high-throughput multiplex bead array as described in Material and Methods. The results are presented in bar charts of median values and interquartile ranges for Z-score of serum concentration (pg/mL). Multiple comparative analysis was performed by Kruskal-Wallis followed by Dunn’s post-test and comparisons between COVID-19 and HC at matching pregnancy trimesters assessed using the Mann-Whitney test. In all cases, significance was considered at p<0.05. Intragroup differences were underscored by the letters “a”, “b” and “c” for comparisons with the 1^st^, 2^nd^ and 3^rd^ trimesters, respectively. Inter-group differences at matching pregnancy trimesters were highlighted denoted by an asterisk (*).

**Figure 2:**
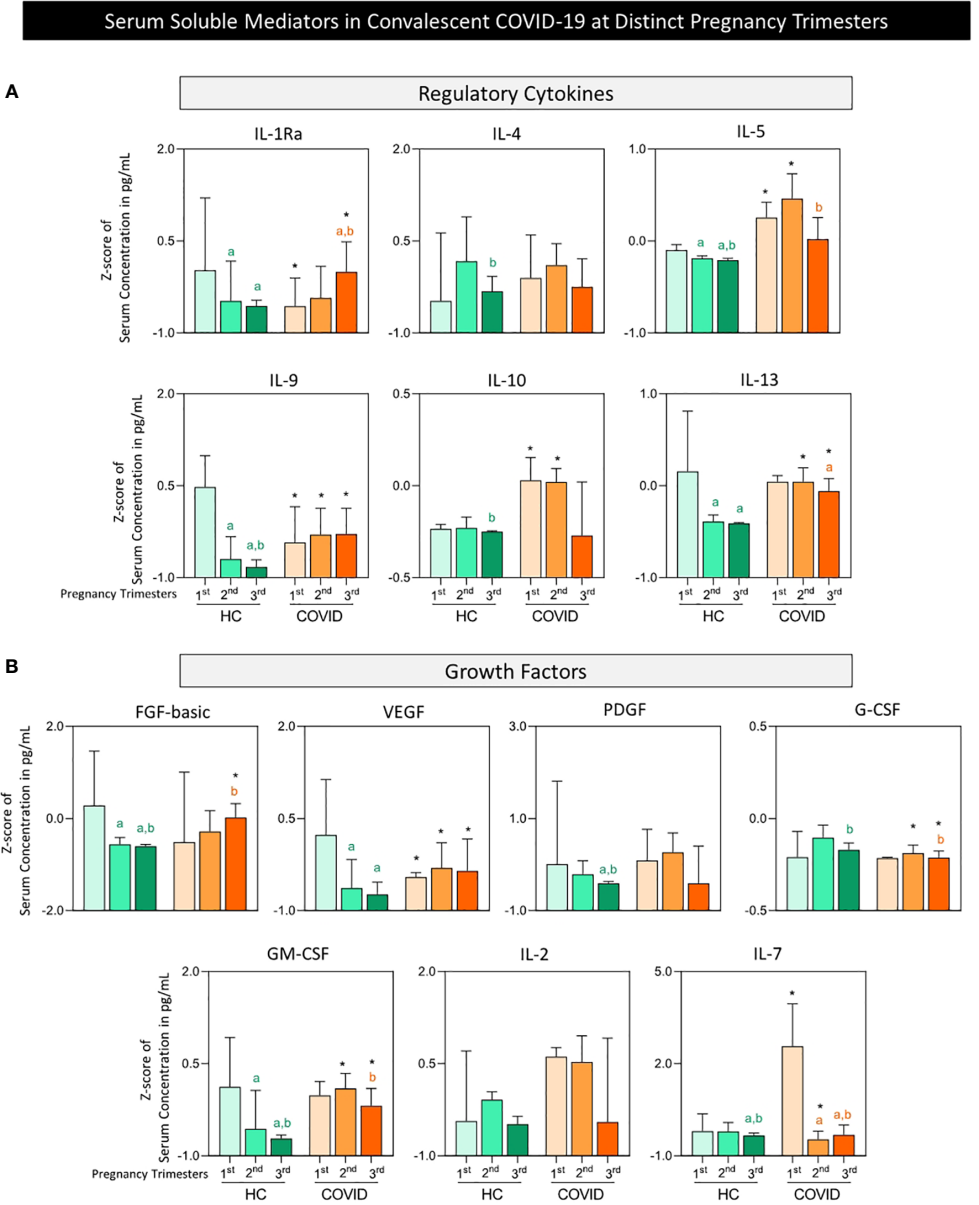
Serum regulatory cytokines and growth factors in convalescent COVID-19 patients at distinct pregnancy trimesters. The levels of: **(A)** regulatory cytokines (IL-1Ra, IL- 4, IL-5, IL-9, IL-10, IL-13), and **(B)** growth factors (FGF-basic, PDGF, VEGF, G-CSF, GM- CSF, IL-2, IL-7) were measured in serum samples from pregnant women with convalescent COVID-19 at 3-20 weeks after symptoms onset (COVID, n=89), with pre-pandemic non-infected pregnant women as a Healthy Control (HC, n=52). The HC and COVID-19 groups were further categorized into subgroups according to pregnancy trimester, referred to as: HC 1^st^ (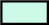, n=21), HC 2^nd^ (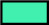, n=10), HC 3^rd^ (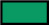, n=21), and COVID 1^st^ (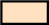, n=7), COVID 2^nd^ (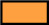, n=34), COVID 3^rd^ (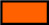, n=48). The measurements were taken by high-throughput multiplex bead array as described in Material and Methods. The results are presented in bar charts of median values and interquartile ranges for Z-score of serum concentration (pg/mL). Multiple comparative analysis was performed by Kruskal-Wallis followed by Dunn’s post-test, and comparisons between COVID-19 and HC at matching pregnancy trimesters assessed by the Mann-Whitney test. In all cases, significance was considered at p<0.05. Intragroup differences were underscored by the letters “a” and “b” for comparisons with the 1^st^ and 2^nd^ trimesters, respectively. Inter-group differences at matching pregnancy trimesters were denoted by an asterisk (*).

The levels of chemokines, pro-inflammatory cytokines, regulatory cytokines and growth factors were measured in serum samples from pregnant women with convalescent COVID-19 at 3-20 weeks after symptoms onset (COVID) and compared with those detected in trimester-matching pre-pandemic non-infected pregnant women as a healthy control (HC). The results are presented in **Figures 1** and **2**.

In general, healthy pregnant women presented a progressive decrease in most serum soluble mediators towards the 2^nd^ and 3^rd^ pregnancy trimester, including: chemokines (CXCL8, CCL11, CCL3 and CCL2); pro-inflammatory cytokines (IL-1β, IL-6, TNF-α, IL-12, IFN-γ, and IL-17); regulatory cytokines (IL-1Ra, IL-4, IL-5, IL-9, IL-10 and IL-13), and growth factors (FGF-basic, VEGF, PDGF, GM-CSF and IL-7). Conversely, progressive increases in CCL4, CCL5, CXCL10 and G-CSF were observed in the HC group. No difference was observed in the HC group for IL-15 and IL-2 (**Figures 1**, **2**).

Overall, higher levels of the most soluble mediators were observed in convalescent COVID-19 pregnant women compared to the healthy controls, especially at the 2^nd^ and 3^rd^ trimesters, including higher levels of CXCL8; CCL11; CCL2; CCL3; IL-1β; IL-6; TNF-α; IL-12; IFN-γ; IL-17; IL-1Ra; IL-5; IL-9; IL-10; IL-13; FGF-basic; VEGF, and GM-CSF. Conversely, lower levels of CCL4, CCL5, CXCL10, G-CSF and IL-7 were observed towards the 2^nd^ and 3^rd^ trimesters in the COVID group compared to the HC group (**Figures 1**, **2**).

Additional analysis amongst the COVID subgroups along the pregnancy trimesters demonstrated an inverted profile of CCL3, IL-1Ra and FGF-basic towards higher levels in the 3^rd^ trimester (**Figures 1**, **2**).

**Supplementary Figure 1** summarizes the major changes observed in serum soluble mediators along the trimesters of healthy and convalescent COVID-19 pregnancy.

### 3.2 Serum soluble mediator signatures in convalescent COVID-19 at distinct pregnancy trimesters

**Figure 3:**
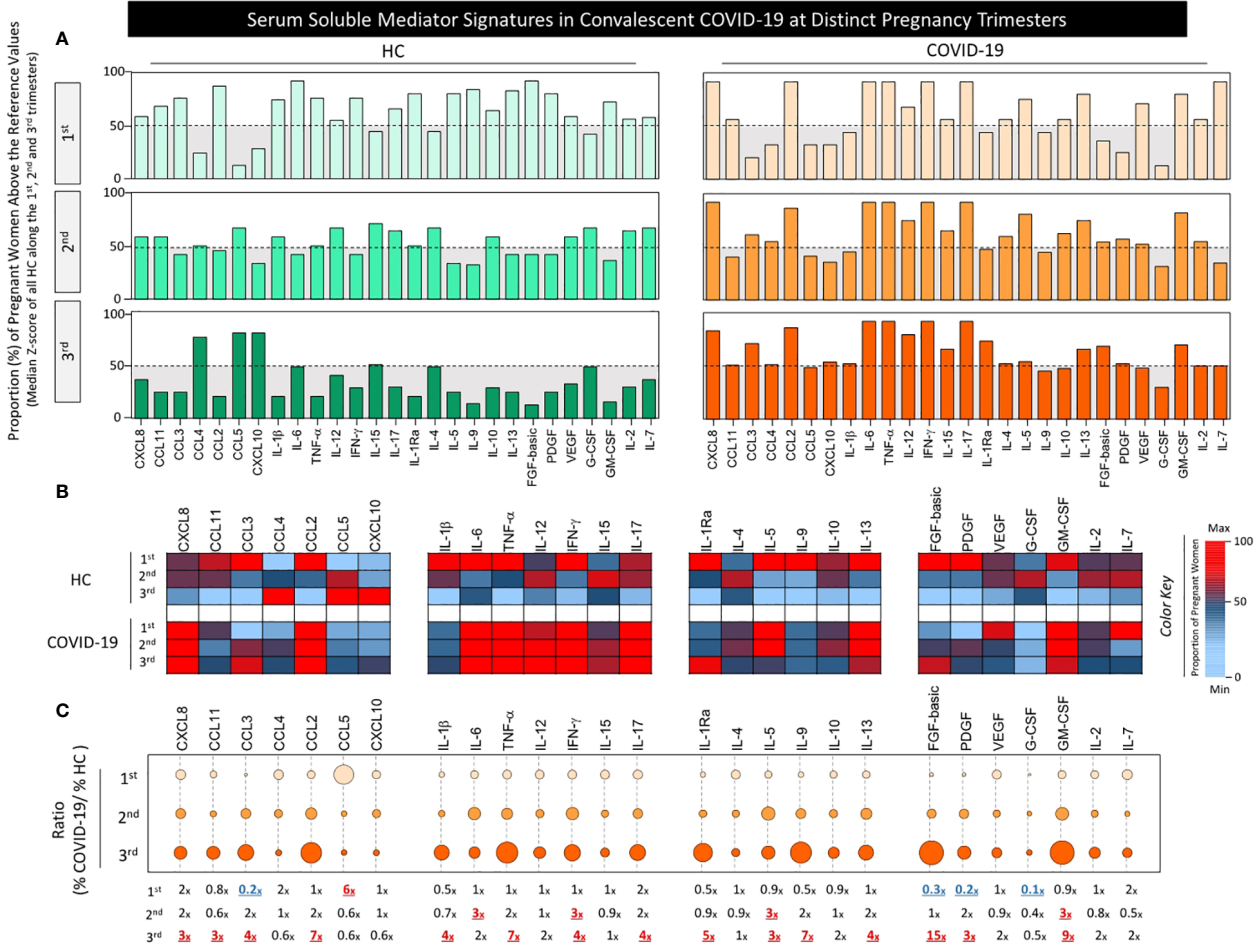
Serum soluble mediator signatures in convalescent COVID-19 patients at distinct pregnancy trimesters. Signatures of: serum chemokines (CXCL8, CCL11, CCL3, CCL4, CCL2, CCL5, and CXCL10), pro-inflammatory cytokines (IL-1β, IL-6, TNF-α, IL-12, IFN-γ, IL-15, and IL-17), regulatory cytokines (IL-1Ra, IL-4, IL-5, IL-9, IL-10, and IL-13), and growth factors (FGF-basic, PDGF, VEGF, G-CSF, GM-CSF, IL-2, and IL-7) were assembled for pregnant women with convalescent COVID-19 at 3-20 weeks after symptoms onset (COVID, n=89) and for pre-pandemic non-infected pregnant women as a Healthy Control (HC, n=52). The HC and COVID-19 groups were further categorized into subgroups according to pregnancy trimester, referred to as: HC 1^st^ (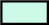, n=21), HC 2^nd^ (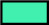, n=10), HC 3^rd^ (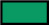, n=21) and COVID 1^st^ (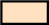, n=7), COVID 2^nd^ (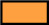, n=34), COVID 3^rd^ (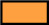, n=48). The measurements were taken by high-throughput multiplex bead array as described in Material and Methods. **(A)** The results are presented in bar charts showing the proportion (%) of pregnant women with serum levels above the reference values (cut-off) defined as the median Z-score of each soluble mediator detected for all HC along the 1^st^, 2^nd^ and 3^rd^ trimesters, as described in Material and Methods. The serum soluble mediators displaying a proportion of pregnant women above 50% (grey zone, dashed line) were included in the set of biomarkers with increased levels. **(B)** Heatmap constructs were further assembled to illustrate the overall profile of serum soluble mediator signatures of COVID and HC subgroups along the pregnancy trimesters. A color key was used to underscore the serum soluble mediators with decreased (proportion <50%, towards light blue), unaltered (proportion =50%, black) or increased levels (proportion>50%, towards red). **(C)** The ratio between the proportion of pregnant women with serum levels above the reference values in the COVID group in relation to HC (%COVID/%HC) was further calculated and presented in orbital graphs. The ratios of each serum soluble mediator along the 1^st^, 2^nd^ and 3^rd^ trimesters are provided in the figure, underscored as decreased (≤0.3x, blue), unaltered (0.4-2x, black) or increased (≥3x, red).

Serum soluble mediator signatures were assembled as the percentage of pregnant women with serum levels above the reference values defined as the median Z-score of each soluble mediator detected in all healthy controls along the pregnancy. The results are presented in **Figure 3**.

Data analysis demonstrated that the proportion of healthy pregnant women with high levels of serum soluble mediators progressively decreased towards the 2^nd^ and 3^rd^ pregnancy trimesters. These data further corroborated that a healthy pregnancy course has a progressive decrease in most serum soluble mediators towards the 2^nd^ and 3^rd^ trimesters, except for CCL4, CCL5 and CXCL10 (**Figure 3A**).

On the other hand, the proportion of pregnant women with convalescent COVID-19 presenting high serum soluble mediator levels progressively increased from the 1^st^ to the 3^rd^ pregnancy trimester (**Figure 3A**). Heatmap constructs further illustrated that the major increase in serum soluble mediators observed in pregnant women with convalescent COVID-19 occurred in pro-inflammatory cytokines, namely IL-6, TNF-α, IL-12, IFN-γ and IL-17 (**Figure 3B**).

The profile of serum soluble mediators was further characterized as the ratio (%COVID/%HC), assessed by dividing the percentage of pregnant women with soluble mediator levels above the reference values observed in the COVID group by the percentage of trimester-matching HC patients. Using this strategy, the results confirmed that a larger set of biomarkers presented a high ratio (%COVID/%HC) towards the 2^nd^ and 3^rd^ trimester. In the 2^nd^ pregnancy trimester, increased ratios were observed for IL-6, IFN-γ, IL-5 and GM-CSF (3x increase) in the COVID-19 group. A larger set of serum soluble mediators with increased ratios was identified for COVID-19 groups at the 3^rd^ pregnancy trimester, including CXCL8, CCL11, IL-5 and PDGF (3x increase), CCL3, IL-1β, IFN-γ, IL-17 and IL-13 (4x increase), CCL2, TNF-α (7x) along with IL-1Ra, IL-9, GM-CSF and FGF-basic (5x, 9x, 9x, and 15x increase, respectively) (**Figure 3C**).

The signatures of serum soluble mediators were also assessed considering the reference values of trimester-matching healthy controls. The results are presented in the **Supplementary Figure 2**. Data reinforce that larger sets of serum soluble mediators with increased ratios were identified for the 2^nd^ and 3^rd^ pregnancy trimesters as compared with trimester-matching controls (**Supplementary Figure 2**).

### 3.3 Serum soluble mediator networks in convalescent COVID-19 at distinct pregnancy trimesers

**Figure 4:**
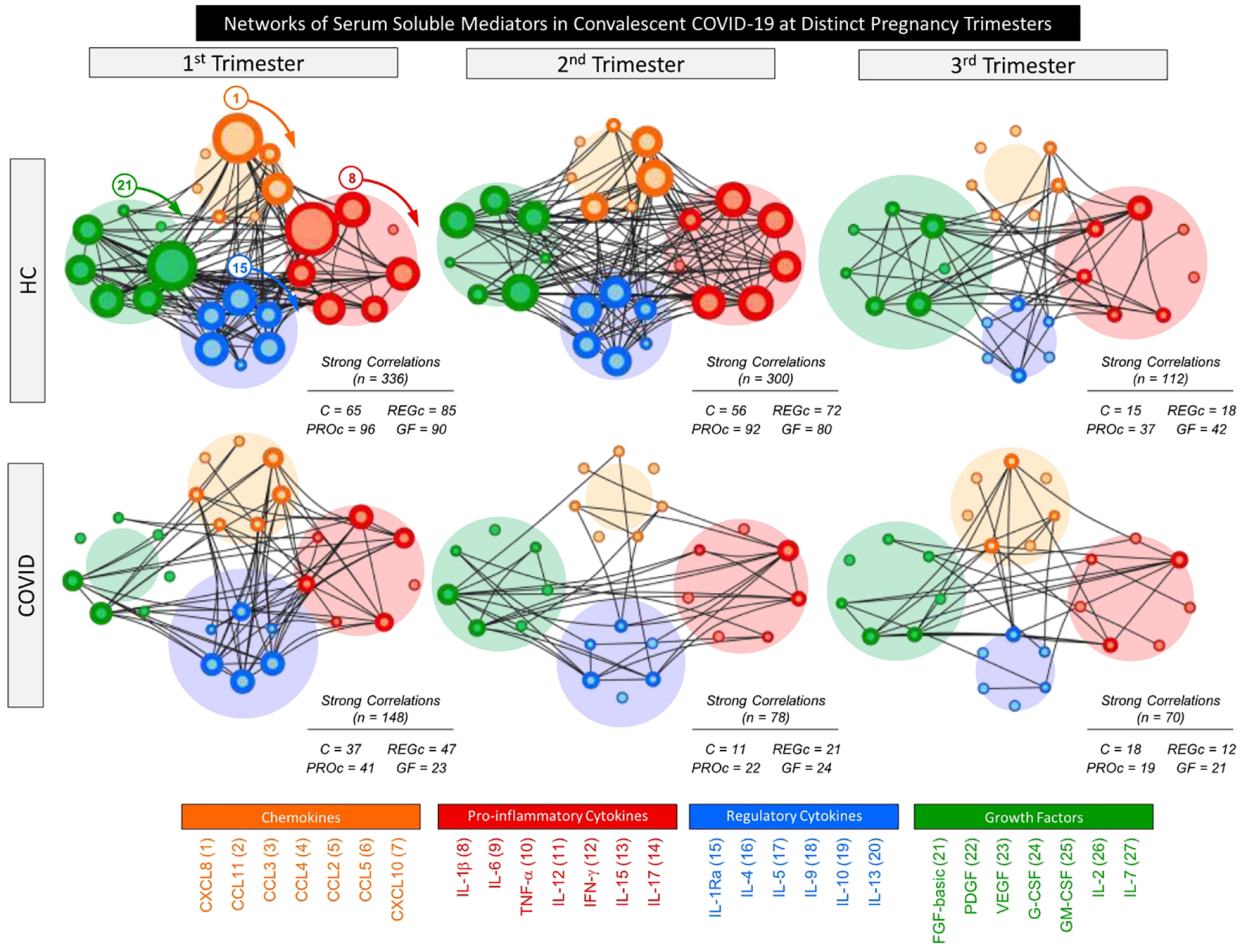
Networks of serum soluble mediators in convalescent COVID-19 patients at distinct pregnancy trimesters. Comprehensive networks were assembled for serum chemokines, pro-inflammatory cytokines, regulatory cytokines, and growth factors observed in pregnant women with convalescent COVID-19 at 3-20 weeks after symptoms onset (COVID, n=89), with pre-pandemic non-infected pregnant women as a Healthy Control (HC, n=52). The HC and COVID-19 groups were further categorized into subgroups according to pregnancy trimester, referred to as: 1^st^ (HC=21 and COVID=7), 2^nd^ (HC=18 and COVID=34) and 3^rd^ (HC=21 and COVID=48). The measurements were taken by high-throughput multiplex bead array as described in Material and Methods. Data analyses were carried out by Pearson and Spearman rank tests with only significant strong correlations (p<0.05 and “r” scores ≥ |0.67|) employed to construct the comprehensive networks. Cluster layout networks were assembled, comprising 4 categories of serum soluble mediators as follows: - Chemokines – C (orange nodes – 1=CXCL8; 2=CCL11; 3=CCL3; 4=CCL4; 5=CCL2, 6=CCL5 and 7=CXCL10); Pro-inflammatory – PROc (red nodes – 8= IL-1β; 9=IL-6; 10= TNF-α; 11=IL-12; 12= IFN-γ; 13=IL-15 and 14=IL-17); Regulatory cytokines – REGc (blue nodes – 15=IL-1Ra; 16=IL-4; 17=IL-5; 18=IL-9; 19=IL-10 and 20=IL-13), and Growth Factors – GF (green nodes – 21=FGF-basic; 22=PDGF; 23=VEGF; 24=GCSF; 25=GM-CSF; 26=IL-2 and 27=IL-7). Node border thickness is proportional to the number of strong correlations. Connecting edges (black lines) are used to link pairs of serum soluble mediators presenting significant correlations. The number of strong correlations (C, PROc, REGc and GF) observed for each network is provided in the figure and used for comparative analysis between COVID and HC, as well as amongst subgroups. The circular background area is proportional to the number of strong correlations of each cluster within the respective network.

Aimed at assessing a panoramic snapshot of serum soluble mediator interplay in pregnant women with convalescent COVID-19 and healthy controls, integrative networks were constructed based on the overall correlation between pairs of molecules. The results are presented in **Figure 4**.

Data analysis demonstrated that healthy pregnancy evolves towards the 3^rd^ trimester with an overall decrease in network connectivity (1^st^ = 336; 2^nd^ = 300 and 3^rd^ = 112 strong correlations). Although pregnant women with convalescent COVID-19 exhibited a similar continuous decrease in network connectivity towards the 3^rd^ trimester (1^st^ = 146; 2^nd^ = 78 and 3^rd^ = 70 strong correlations), the number of connections was remarkably lower in the COVID group compared to HC group (**Figure 4**).

Overall, the analysis of cluster connectivity during healthy pregnancy showed that pro-inflammatory cytokines presented more connections at the 1^st^ and 2^nd^ trimesters (96 and 92 strong correlations, respectively), with growth factor predominance at the 3^rd^ trimester (42 strong correlations). Conversely, the COVID group displayed a predominance of regulatory cytokines in the 1^st^ trimester (47 strong correlations) with growth factor predominance in the 2^nd^ and 3^rd^ trimesters (24 and 21, respectively) (**Figure 4**).

In general, convalescent COVID-19 infection during pregnancy leads to a loss of network connectivity, with fewer strong correlations and changes in the predominance of connectivity amongst the categories of serum soluble mediators (**Figure 4**).

### 3.4 Descriptive analysis of serum soluble mediator networks in convalescent COVID-19 patients at distinct pregnancy trimesters

**Figure 5:**
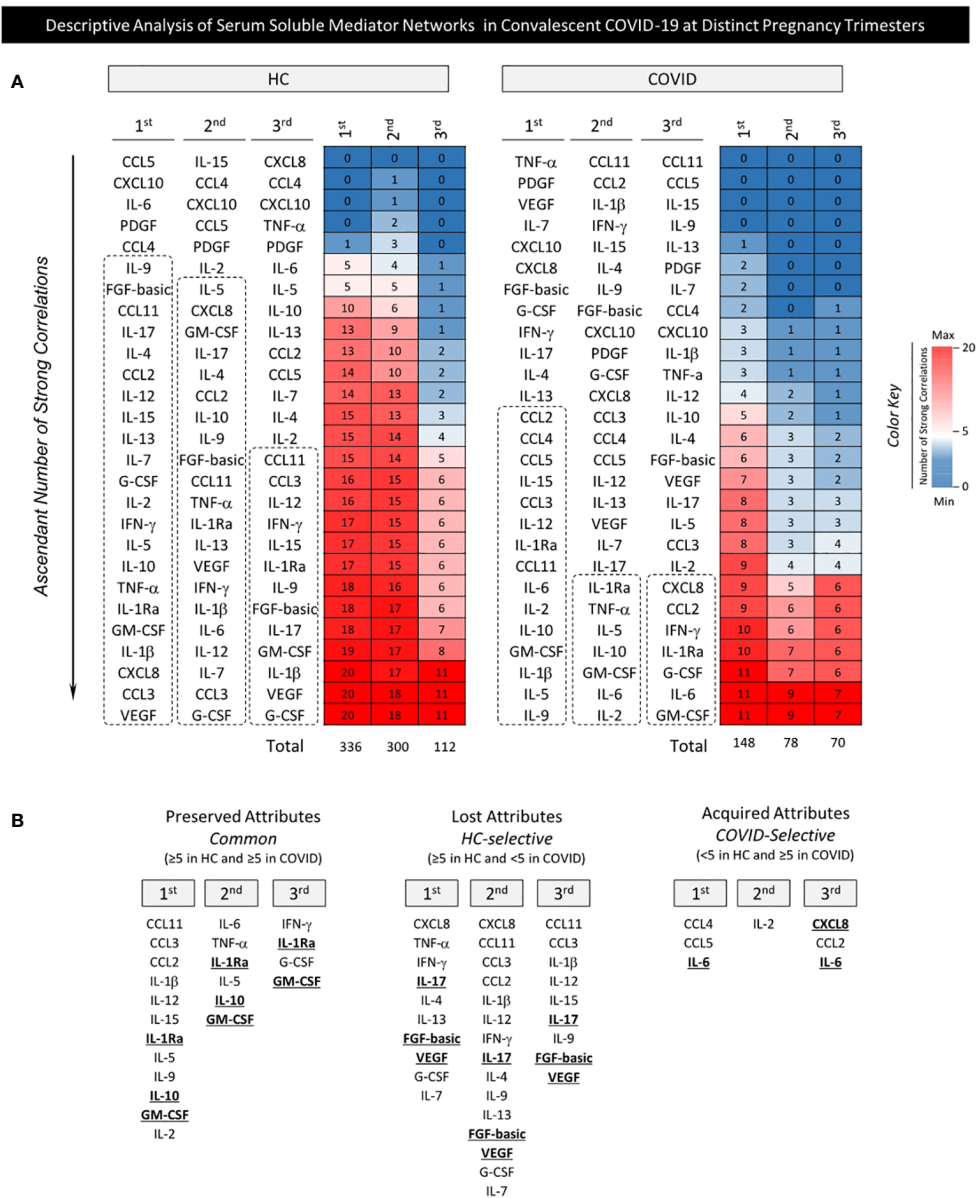
Descriptive analysis of serum soluble mediator networks in convalescent COVID-19 patients at distinct pregnancy trimesters. The overall profile of serum soluble mediator networks was assessed in pregnant women with convalescent COVID-19 at 3-20 weeks after symptoms onset (COVID, n=89) with pre-pandemic non-infected pregnant women as a Healthy Control (HC, n=52). The HC and COVID-19 groups were further categorized into subgroups according to pregnancy trimester, referred to as: 1^st^ (HC=21 and COVID=7), 2^nd^ (HC=10 and COVID=34), and 3^rd^ (HC=21 and COVID=48). **(A)** The ascendant number of strong correlations was arranged to identify the set of biomarkers with five or more (≥ 5) strong correlations with other molecules at each pregnancy trimester in HC and COVID subgroups. Heatmap constructs were assembled to illustrate the overall profile of serum soluble mediator networks of COVID and HC subgroups along the pregnancy trimesters. A color key was used to underscore the serum soluble mediators with ≥ 5 strong correlations (towards red). **(B)** A summary of preserved (≥5 in HC and ≥5 in COVID), lost (≥5 in HC and <5 in COVID) or acquired (<5 in HC and ≥5 in COVID) attributes were identified by Venn diagram analysis. Attributes identified along the trimesters are highlighted by bold underline format.

In order to provide a more comprehensive overview of the network connectivity between serum soluble mediators in pregnant women with convalescent COVID-19 and healthy controls along the pregnancy trimesters, a descriptive Venn diagram analysis was performed to identify the set of biomarkers with preserved (common), lost or acquired (selective) attributes with five or more (≥ 5) strong correlations in COVID subgroups as compared to the trimester-matching HC group. The results are presented in the **Figure 5**.

Heatmap constructs were assembled to organize the serum soluble mediators with an ascending order of strong correlations and identify the set of biomarkers with five or more (≥ 5) strong correlations at each pregnancy trimester in the COVID and HC groups (**Figure 5A**).

Data analysis demonstrated that the number of preserved attributes referred to as common in HC and COVID (≥5 strong correlations in HC and COVID) with five or more correlations progressively decreased from the 1^st^ (n=12) to the 2^nd^ (n=6) and 3^rd^ trimesters (n=4). In detail: 1^st^: CCL11, CCL3, CCL2, IL-1β, IL-12, IL-15, IL-1Ra, IL-5, IL-9, IL-10, GM-CSF, and IL-2; 2^nd^: IL-6, TNF-α, IL-1Ra, IL-5, IL-10, GM-CSF and 3^rd^:IFN-γ, IL-1Ra, G-CSF, and GM-CSF.

The number of lost attributes referred to as HC-selective (≥5 strong correlations in HC and <5 strong correlations in COVID) was higher in the 2^nd^ trimester (n=15) compared to 1^st^ (n=10) and 3^rd^ (n=9). In detail: 1^st^: CXCL8, TNF-α, IFN-γ, IL-17, IL-4, IL-13, FGF-basic, VEGF, G-CSF, and IL-7; 2^nd^: CXCL8, CCL11, CCL3, CCL2, IL-1β, IL-12, IFN-γ, IL-17, IL-4, IL-9, IL-13, FGF-basic, VEGF, G-CSF, and IL-7; 3^rd^: CCL11, CCL3, IL-1β, IL-12, IL-15, IL-17, IL-9, FGF-basic, and VEGF. A set of acquired attributes, named COVID-selective (<5 strong correlations in HC and ≥5 strong correlations in COVID) were identified in each trimester: 1^st^ (n=3): CCL4, CCL5, and IL-6, 2^nd^: (n=1) IL-2, and 3^rd^ (n=3) CXCL8, CCL2 and IL-6 (**Figure 5B**).

From an overall perspective, a pronounced decrease in network connectivity between serum soluble mediators was observed in convalescent COVID-19 infection during pregnancy as demonstrated by the fewer number of molecules establishing strong correlations driven by an imbalance between preserved, lost and acquired attributes in the COVID group. While IL-1Ra, IL-10 and GM-CSF presented a preserved number of correlations (≥5 strong correlations in HC and COVID), IL-17, FGF-basic and VEGF lost connectivity throughout pregnancy. IL-6 (at 1^st^ and 3^rd^ trimesters) and CXCL8 (at 3rd trimester) were included in a set of acquired attributes, named COVID-selective (≥5 strong correlations in COVID and <5 in HC) (**Figure 5B**, bold underline attributes).

### 3.5 Multivariate analysis of serum soluble mediators in convalescent COVID-19 patients at distinct pregnancy trimesters

**Figure 6:**
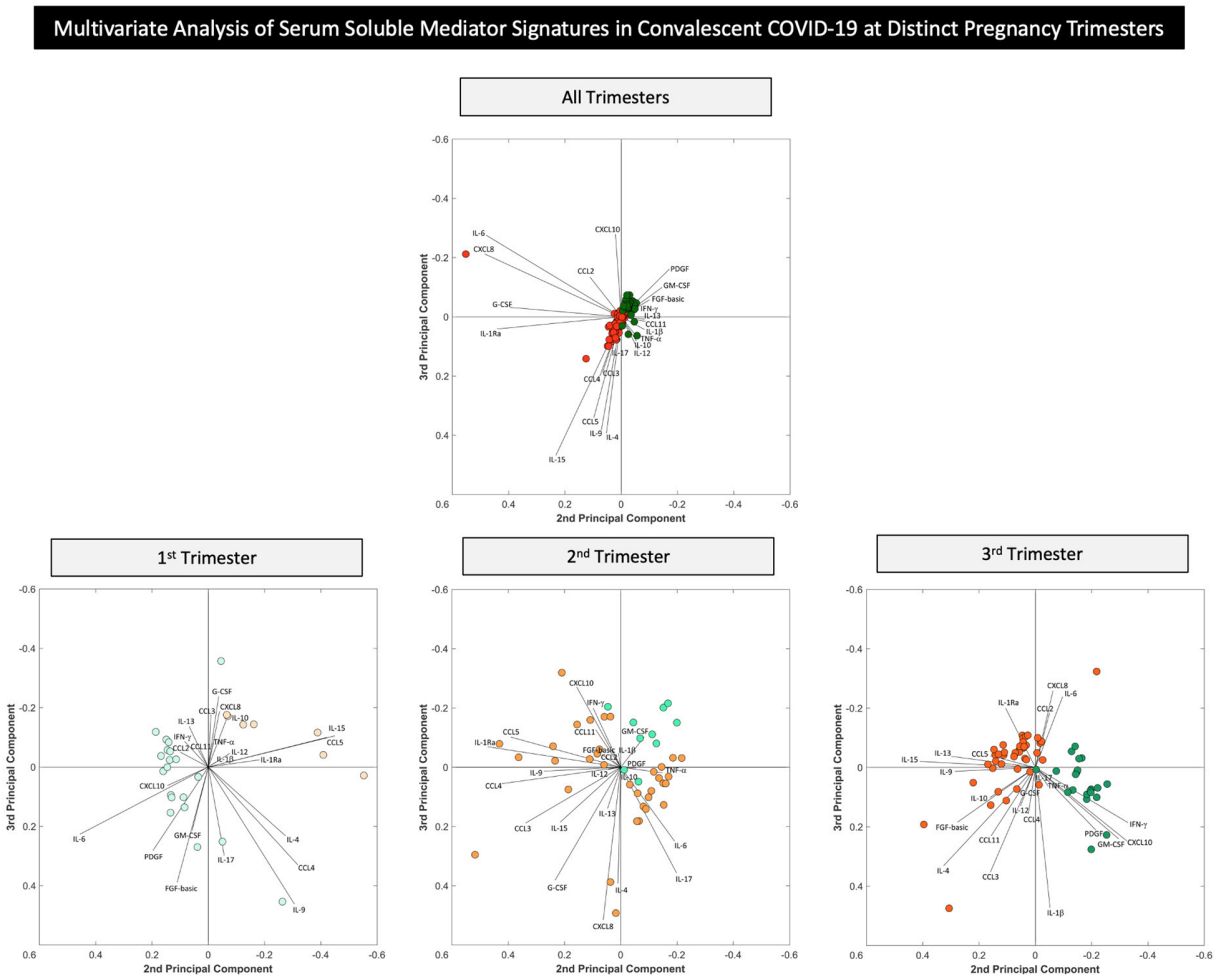
*Multivariate analysis of serum soluble mediator networks in convalescent COVID-19 patients at distinct pregnancy trimesters.* Multivariate analysis of serum chemokines, pro-inflammatory cytokines, regulatory cytokines and growth factors were performed to verify the ability of soluble mediators to cluster convalescent COVID-19 pregnant women at 3-20 weeks after symptoms onset (COVID, n=89), with pre-pandemic non-infected pregnant women as a Healthy Control (HC, n=52) as well as subgroups of COVID-19 and HC categorized according to pregnancy trimester, referred to as: HC 1^st^ (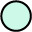, n=21), HC 2^nd^ (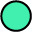, n=10), HC 3^rd^ (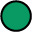, n=21) and COVID 1^st^ (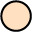, n=7), COVID 2^nd^ (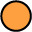, n=34), COVID 3^rd^ (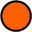, n=48). The measurements were taken by high-throughput multiplex bead array as described in Material and Methods. Principal Component Analysis (PCA) was carried out by MATLAB software as described in Material and Methods. PCA coordinates (2^nd^ and 3^rd^ principal components) were used to compare and visualize the grouping of convalescent COVID-19 *vs* HC and subgroups according to pregnancy trimesters.

Multivariate analysis of chemokines, pro-inflammatory cytokines, regulatory cytokines and growth factors was performed using PCA to verify the ability of serum mediators to cluster convalescent COVID-19 pregnant women apart from trimester-matching pre-pandemic non-infected pregnant women as a healthy control (HC). The results are presented in **Figure 6**. The PCA coordinates (2^nd^ and 3^rd^ principal components) demonstrated that although convalescent COVID pregnant women could be clustered apart from the HC when considering all trimesters together, the segregation profile was more evident when the COVID and HC subgroups were compared at matching gestational trimesters (**Figure 6**). Vector analysis conducted in the 1^st^ trimester indicated that CXCL8, CCL3, CCL5, IL-1β, TNF-α, IL-12, IL-15, IL-1Ra, IL-10, and G-CSF were associated with convalescent COVID-19 in pregnant women. Data from the 2^nd^ trimester showed that most soluble mediators were vectors associated with differential distribution of convalescent COVID-19 in pregnant women, except for GM-CSF. Additionally, the PCA coordinates obtained from the 3^rd^ trimester demonstrated that several soluble mediators were vectors related to convalescent COVID-19 in pregnant women, except for CXCL10, IL-1β, TNF-α, IFN-γ, PDGF and GM-CSF (**Figure 6**).

## 4 Discussion

Pregnancy triggers a unique immunological status, aiming to protect the fetus from maternal rejection and guarantee fetal development until birth. Several studies have reported that the immune system plays a balancing role during pregnancy with constant changes according to maternal and fetal demands (16, 17). Physiological changes in immune status during pregnancy are often characterized by alterations in cell-mediated immunity and humoral responses, from the 1^st^ to 3^rd^ pregnancy trimesters. Previous studies have demonstrated that successful implantation is associated with a transient increase in systemic proinflammatory profile followed by a switch toward an anti-inflammatory profile after blastocyst transfer when pregnancy is confirmed (18). Pregnant women are particularly susceptible to COVID-19 due to physiological changes in the immune system, which may result in an altered response to SARS-CoV-2 infection in pregnancy. Furthermore, SARS-CoV-2 infection during pregnancy can disrupt the immune response homeostasis, impacting the maternal immune activation, with effects on pregnancy outcome and offspring (12, 13, 19, 20). It has already been reported that the adverse impacts of the COVID-19 pandemic on maternal health are not limited to morbidity and mortality caused by the disease itself, but are also associated with adverse pregnancy outcomes, including preeclampsia, preterm birth and stillbirth (19).

Most of the information on the impact of SARS-CoV-2 infection in pregnancy has been derived from reports concerning acute symptomatic infection (21). However, little data is available regarding the long-term impact of SARS-CoV-2 infection on pregnancy during the convalescent phase of the disease. In view of this, we designed this study as a pioneer exploratory investigation to perform descriptive and panoramic analysis of serum soluble mediator interplay in pregnant women during the convalescent phase of SARS-CoV-2 infection throughout prenatal care. This study comprises an innovative investigation of the long-lasting impact of SARS-CoV-2 infection during pregnancy focusing on the analysis of the immune response during the convalescent phase comprising 3-20 weeks after symptoms onset.

Our results demonstrate that, in general, serum soluble mediators have different trajectories during healthy pregnancy and are disturbed in pregnant women with convalescent SARS-CoV-2 infection. Herein, healthy pregnant women presented a progressive decrease in most serum soluble mediators towards the 2^nd^ and 3^rd^ pregnancy trimester, including chemokines, pro-inflammatory and regulatory cytokines, in addition to growth factors. Previous studies corroborate our findings in healthy pregnancies. The levels of chemokines and pro-inflammatory cytokines usually peak in the first trimester of pregnancy and decline in the 2^nd^ and 3^rd^ trimesters, while regulatory cytokines and growth factors have diverse trajectories (22, 23). Our findings highlighted that higher levels of most soluble mediators were observed in the COVID group compared to HC control group. The major increase occurred in pro-inflammatory cytokines, including IL-6, TNF-α and IFN-γ, a larger set of biomarkers with elevated COVID/HC ratios observed towards the 2^nd^ (3x increase) and 3^rd^ (3-15x increase) pregnancy trimesters. Studies of immune mediators in SARS-CoV-2 infection during pregnancy remain scarce. It has been proposed that the immunomodulation observed during pregnancy may protect pregnant COVID-19 patients from suffering from a cytokine storm (15, 24). However, no studies focusing on comparative analysis of immunological profiles of COVID-19 and healthy pregnant women at matching pregnancy trimesters have been reported. It is noteworthy that due to physiological changes in the immune response during pregnancy, the inclusion of trimester-matching healthy controls is essential to enable conclusive analysis. Therefore, our study is pioneering in terms of providing a detailed profile of long-lasting changes during convalescent COVID-19 infection in pregnancy as it made direct comparison to trimester-matching healthy controls. Our findings did not support that an immunomodulatory profile is triggered by SARS-CoV-2 infection during pregnancy. In fact, the lower levels of soluble mediators previously reported in convalescent COVID-19 pregnant women were compared with those observed in non-pregnant women and did not consider the physiological changes triggered by pregnancy or alterations in soluble mediators inherent in trimesters (24). Moreover, other studies proposing the immunomodulatory state for acute COVID-19 infection during pregnancy in comparison to healthy pregnant women did not consider stratification by gestational trimesters (15). According to our findings, higher levels of serum soluble mediators were observed for convalescent COVID-19 infection during pregnancy, especially IL-6, TNF-α and IFN-γ in comparison to healthy pregnant women at matching pregnancy trimesters.

Successful pregnancy requires finely coordinated communication between the maternal and fetal microenvironments. Cytokine signaling pathways participate as mediators of these communications to guarantee healthy pregnancy. From the existing data available, there is no consensus trend for the changes observed for IL-6, TNF-α and IFN-γ during pregnancy (16). Several studies have demonstrated that IL-6, TNF-α and IFN-γ concentrations significantly increased between the 1^st^, 2^nd^ and 3^rd^ trimesters of healthy pregnancy (25–30). However, corroborating our findings, other authors detected significant reductions in IL-6, TNF-α and IFN-γ in maternal serum concentrations between the 1^st^ and 3^rd^ trimesters (31–33).

Infections or inflammatory conditions, such as COVID-19 during pregnancy, can have a detrimental impact on fetal development and also contribute to pregnancy-associated pathological conditions (34). Despite the conflicting data regarding the overall profile of IL-6, TNF-α and IFN-γ during healthy pregnancy, there is a consensus that the establishment of a pro-inflammatory microenvironment is associated with the risk of developing pregnancy-associated pathological conditions, including pregnancy loss, preeclampsia, and gestational diabetes mellitus (35). In this sense, the upregulation of pro-inflammatory cytokines in pregnant women with convalescent COVID-19 may suggest that these patients are more vulnerable to developing adverse pregnancy outcomes.

Integrative network analysis demonstrated that both HC and convalescent COVID-19 pregnancies evolve with decreasing connectivity between serum soluble mediators towards the 3^rd^ trimester. However, the COVID group exhibited a remarkably lower number of connections. Overall, IL-1Ra, IL-10 and GM-CSF presented a preserved number of correlations throughout the pregnancy.

Further research is warranted to determine the precise IL-10 profile during healthy pregnancy (14). A few studies have reported that IL-10 significantly increases from the 1^st^ to the 2^nd^ and 3^rd^ trimesters in healthy pregnancy (30, 36). However, corroborating our findings, other studies have detected that IL-10 decreases between the 1^st^ and 3^rd^ trimesters (31, 37). Considering the critical role of IL-10 as a chief anti-inflammatory cytokine, the preserved IL-10 connectivity axis observed during the 1^st^ and 2^nd^ trimesters may represent a mechanism to protect the fetus from maternal pro-inflammatory rejection and guarantee fetal development until birth.

Our data demonstrated that IL-1Ra decreased in convalescent COVID-19 pregnant women in the 1^st^ trimester but increased in the 3^rd^ trimester. Previous studies reported that IL-1Ra levels increased during the inflammatory response to control acute inflammation and prevent immunopathological events (38). The IL-1 receptor antagonist (IL-1Ra) is an anti-inflammatory cytokine that blocks IL-1α and IL-1β functions and modulates their biological effects (39). It has been previously demonstrated in experimental models that high IL-1Ra levels at the beginning of pregnancy may lead to miscarriage due to impaired embryonic adhesion (40), and data from human studies showed that higher levels of circulating IL-1Ra have been reported in adverse pregnancy outcomes, including preeclampsia (41). Regarding the changes in IL-1Ra levels observed in convalescent COVID-19 along the pregnancy trimesters, our findings of preserved correlation profile between IL-1Ra and other soluble mediators throughout the pregnancy may suggest that an intricate microenvironment of soluble mediators is relevant to guarantee fetal development until birth.

Our data also demonstrated that GM-CSF presented preserved correlation with other soluble mediators throughout pregnancy. It was previously reported that after embryo implantation, GM-CSF participates in a network of cytokines and growth factors that regulate morphological and functional development of the placenta (42).

Conversely, despite increases in IL-17, FGF-basic and VEGF, loss of connectivity was observed throughout pregnancy. IL-17 up-regulates the expression of a variety of biological molecules with angiogenic properties including VEGF (43–47). VEGF plays a central role in vasculogenesis and angiogenesis, which augments vascular endothelial cell proliferation, migration, and survival. Moreover, data from previous studies have shown that IL-17 can induce placental oxidative stress and vascular dysfunction, resulting in hypertension and increased risk of preeclampsia (48). The loss of network connection of IL-17 and VEGF with other soluble mediators throughout the pregnancy may lead to intrinsic vascular dysfunction that results in impaired neonatal development. Post-natal follow-up studies may contribute to identifying impaired new-born growth and development related to altered angiogenesis.

Our data also demonstrated that IL-6 and CXCL8 were included in the set of attributes acquiring strong correlation in the 3^rd^ pregnancy trimester, named COVID-selective correlations. Implications of IL-6 and CXCL8 in pregnancy-associated pathological conditions, such as pregnancy loss, preeclampsia, gestational diabetes mellitus, and infection/inflammation have been reported (35). These two soluble mediators are abundantly produced at the feto-maternal interface throughout pregnancy and have been shown to participate in several pregnancy-related events. Unbalanced expression/secretion of IL-6 and CXCL8 at the feto-maternal interface has been indicated in unexplained pregnancy loss (35). A study of the dynamic connections of the soluble mediator network in pre-eclampsia identified positive correlation between IL-6 and CXCL8, suggesting that these molecules are implicated in the pathophysiology of this pregnancy-associated disease (35, 49). Moreover, a meta-analysis and systematic review suggested a role of CXCL8 in shaping the immune microenvironment in gestational diabetes mellitus (50).

The present study has some limitations. The low number of pregnant women enrolled in each pregnancy trimester re-enforce the need to further validate our findings. This work was performed during circulation of the B.1.1.28 and B.1.1.33 SARS-CoV-2 strains and therefore, the impact of other variants on the immunological profiles remains to be addressed. Despite the pioneering approach of this exploratory investigation, the observational design with multiple comparisons without corrections for co-morbidities or other confounding variables also constitutes a study limitation that may interfere in the levels of systemic soluble mediators. Moreover, regardless the relevance of nutritional aspects ant the dietary inflammatory indices interfering in the immune response during pregnancy (51), we did not have the opportunity to address this issue in the present investigation.

In conclusion, the main finding of this study, a pronounced increase in serum levels of soluble mediators with decreased network interplay between them, portrayed an imbalanced immune response in convalescent COVID-19 infection during pregnancy that may contribute to the prevention or management of clinical course pregnancy complications.

## Data availability statement

The original contributions presented in the study are included in the article/**Supplementary Material**. Further inquiries can be directed to the corresponding authors.

## Ethics statement

The studies involving human participants were reviewed and approved by National Commission for Ethics in Research in Brazil (CONEP, CAAE 32359620.0.0000.5558). The patients/participants provided their written informed consent to participate in this study.

## Author contributions

Study design: GF, LS, FM, CA, KC, MC, RT, ON, AS, CPA, AZ and LM. Advisory committee: CG, DM-S, PK, ON, LC, COA, and AZ. Funding acquisition: LE, OM-F and LM. Sample collection, experimental procedures, and data acquisition: ÂS, AC, YP, DA, LL, RN, PA, LG, LD, and JCdR. Data analysis: GF, LS, GJ-S, HS, LA, MG, PB, JBdS, IC-R, AC-A, VP-M, AC, and OM-F. Writing and reviewing the manuscript: GF, LS, GJ-S, HS, OM-F and LM. All authors contributed to the article and approved the submitted version.

## References

[1] World Health Organization. WHO coronavirus (COVID-19) dashboard data (2022). Available at: https://covid19.who.int/ (Accessed January 25, 2022).

[2] Brandt JS Hill J Reddy A Schuster M Patrick HS Rosen T. Epidemiology of coronavirus disease 2019 in pregnancy: risk factors and associations with adverse maternal and neonatal outcomes. Am J Obstet Gynecol (2021) 224(4):e1–9. doi: 10.1016/j.ajog.2020.09.043 PMC7518835 32986989

[3] Khoury R Bernstein PS Debolt C Stone J Sutton DM Simpson LL. Characteristics and outcomes of 241 births to women with severe acute respiratory syndrome coronavirus 2 (SARS-CoV-2) infection at five new York city medical centers. Obstetrics Gynecol (2020) 136(2):273–82. doi: 10.1097/AOG.0000000000004025 32555034

[4] Metz TD Clifton RG Hughes BL Sandoval G Saade GR Grobman WA. Disease severity and perinatal outcomes of pregnant patients with coronavirus disease 2019 (COVID-19). Obstetrics Gynecol (2021) 137(4):571–80. doi: 10.1097/AOG.0000000000004339 PMC7984765 33560778

[5] Darif D Hammi I Kihel A Idrissi Saik I Guessous F Akarid K. The pro-inflammatory cytokines in COVID-19 pathogenesis: What goes wrong? Microb Pathog (2021) 153:104799. doi: 10.1016/j.micpath.2021.104799 33609650 PMC7889464

[6] Asakura H Ogawa H. COVID-19-associated coagulopathy and disseminated intravascular coagulation. Int J Hematol (2021) 113(1):45–57. doi: 10.1007/s12185-020-03029-y 33161508 PMC7648664

[7] Chen R Lan Z Ye J Pang L Liu Y Wu W. Cytokine storm: The primary determinant for the pathophysiological evolution of COVID-19 deterioration. Front Immunol (2021) 12. doi: 10.3389/fimmu.2021.589095 PMC8115911 33995341

[8] Allotey J Fernandez S Bonet M Stallings E Yap M Kew T. Clinical manifestations, risk factors, and maternal and perinatal outcomes of coronavirus disease 2019 in pregnancy: living systematic review and meta-analysis. BMJ (2020), 370:m3320. doi: 10.1136/bmj.m3320 32873575 PMC7459193

[9] Villar J Ariff S Gunier RB Thiruvengadam R Rauch S Kholin A. Maternal and neonatal morbidity and mortality among pregnant women with and without COVID-19 infection. JAMA Pediatr (2021) 175(8):817. doi: 10.1001/jamapediatrics.2021.1050 33885740 PMC8063132

[10] Huntley BJF Huntley ES di Mascio D Chen T Berghella V Chauhan SP. Rates of maternal and perinatal mortality and vertical transmission in pregnancies complicated by severe acute respiratory syndrome coronavirus 2 (SARS-Co-V-2) infection. Obstetrics Gynecol (2020) 136(2):303–12. doi: 10.1097/AOG.0000000000004010 32516273

[11] Savasi VM Parisi F Patanè L Ferrazzi E Frigerio L Pellegrino A. Clinical findings and disease severity in hospitalized pregnant women with coronavirus disease 2019 (COVID-19). Obstetrics Gynecol (2020) 136(2):252–8. doi: 10.1097/AOG.0000000000003979 32433453

[12] Cavalcante MB Cavalcante CTMB Sarno M Barini R Kwak-Kim J. Maternal immune responses and obstetrical outcomes of pregnant women with COVID-19 and possible health risks of offspring. J Reprod Immunol (2021) 143:103250. doi: 10.1016/j.jri.2020.103250 33249335 PMC7676367

[13] Cavalcante MB de Melo Bezerra Cavalcante CT Cavalcante ANM Sarno M Barini R Kwak-Kim J. COVID-19 and miscarriage: From immunopathological mechanisms to actual clinical evidence. J Reprod Immunol (2021) 148:103382. doi: 10.1016/j.jri.2021.103382 34534878 PMC8429080

[14] Parisi F Milazzo R Savasi VM Cetin I. Maternal low-grade chronic inflammation and intrauterine programming of health and disease. Int J Mol Sci (2021) 22(4):1732. doi: 10.3390/ijms22041732 33572203 PMC7914818

[15] Fernandes GM Motta F Sasaki LMP da Silva ÂP Miranda AM de Carvalho AO. Pregnancy outcomes and child development effects of SARS-CoV-2 infection (PROUDEST trial): Protocol for a multicenter, prospective cohort study. JMIR Res Protoc (2021) 10(4):e26477. doi: 10.2196/26477 33793409 PMC8059788

[16] Spence T Allsopp PJ Yeates AJ Mulhern MS Strain JJ McSorley EM. Maternal serum cytokine concentrations in healthy pregnancy and preeclampsia. J Pregnancy (2021) 2021:1–33. doi: 10.1155/2021/6649608 PMC7925069 33680514

[17] Chen G Liao Q Ai J Yang B Bai H Chen J. Immune response to COVID-19 during pregnancy. Front Immunol (2021) 12. doi: 10.3389/fimmu.2021.675476 PMC8126657 34012458

[18] Zhao Y Zhang T Guo X Wong CK Chen X Chan YL. Successful implantation is associated with a transient increase in serum pro-inflammatory cytokine profile followed by a switch to anti-inflammatory cytokine profile prior to confirmation of pregnancy. Fertile Sterile (2021) 115(4):1044–53. doi: 10.1016/j.fertnstert.2020.10.031 33272613

[19] Wei SQ Bilodeau-Bertrand M Liu S Auger N. The impact of COVID-19 on pregnancy outcomes: a systematic review and meta-analysis. Can Med Assoc J (2021) 193(16):E540–8. doi: 10.1503/cmaj.202604 PMC8084555 33741725

[20] Chen G Zhang Y Zhang Y Ai J Yang B Cui M. Differential immune responses in pregnant patients recovered from COVID-19. Signal Transduct Target Ther (2021) 6(1):289. doi: 10.1038/s41392-021-00703-3 34326311 PMC8320317

[21] Mullins E Perry A Banerjee J Townson J Grozeva D Milton R. Pregnancy and neonatal outcomes of COVID-19: The PAN-COVID study. Eur J Obstetrics Gynecol Reprod Biol (2022) 276:161–7. doi: 10.1016/j.ejogrb.2022.07.010 PMC9295331 35914420

[22] Jarmund AH Giskeødegård GF Ryssdal M Steinkjer B Stokkeland LMT Madssen TS. Cytokine patterns in maternal serum from first trimester to term and beyond. Front Immunol (2021) 12. doi: 10.3389/fimmu.2021.752660 PMC8552528 34721426

[23] Azizieh F Dingle K Raghupathy R Johnson K VanderPlas J Ansari A. Multivariate analysis of cytokine profiles in pregnancy complications. Am J Reprod Immunol (2018) 79(3):e12818. doi: 10.1111/aji.12818 29450942 PMC5838769

[24] Tartaglia E Bordoni V Oliva A Vergori A Girardi E Antinori A. T Helper profile in pregnant women recovered from COVID-19. J Reprod Immunol (2022) 153:103661. doi: 10.1016/j.jri.2022.103661 35839525 PMC9258427

[25] Subha M Pal P Pal GK Habeebullah S Adithan C Sridhar MG. Decreased baroreflex sensitivity is linked to sympathovagal imbalance, low-grade inflammation, and oxidative stress in pregnancy-induced hypertension. Clin Exp Hypertens (2016) 38(8):666–72. doi: 10.1080/10641963.2016.1200596 27935325

[26] Blackmore ER Moynihan JA Rubinow DR Pressman EK Gilchrist M O’Connor TG. Psychiatric symptoms and proinflammatory cytokines in pregnancy. Psychosom Med (2011) 73(8):656–63. doi: 10.1097/PSY.0b013e31822fc277 PMC3188677 21949424

[27] Simavli S Derbent AU Uysal S Turhan NÖ. Hepcidin, iron status, and inflammation variables among healthy pregnant women in the Turkish population. J Maternal-Fetal Neonatal Med (2014) 27(1):75–9. doi: 10.3109/14767058.2013.804054 23662610

[28] Björkander S Bremme K Persson JO van Vollenhoven RF Sverremark-Ekström E Holmlund U. Pregnancy-associated inflammatory markers are elevated in pregnant women with systemic lupus erythematosus. Cytokine (2012) 59(2):392–9. doi: 10.1016/j.cyto.2012.04.046 22633082

[29] Lindsay K Buss C Wadhwa P Entringer S. Maternal stress potentiates the effect of an inflammatory diet in pregnancy on maternal concentrations of tumor necrosis factor alpha. Nutrients (2018) 10(9):1252. doi: 10.3390/nu10091252 30200631 PMC6163870

[30] Olimpia SS Magdalena P Tomasz P Piotr W Elzbieta RW. Changes in the concentration of sHLA-I and selected cytokines in pregnancy complicated by antiphospholipid syndrome. Ginekol Pol (2011) 82(5):354–8. doi: 10.3390/nu10091252 21851034

[31] Stokkeland LMT Giskeødegård GF Stridsklev S Ryan L Steinkjer B Tangerås LH. Serum cytokine patterns in first half of pregnancy. Cytokine (2019) 119:188–96. doi: 10.1016/j.cyto.2019.03.013 30954016

[32] Doria A Cutolo M Ghirardello A Zen M Villalta D Tincani A. Effect of pregnancy on serum cytokines in SLE patients. Arthritis Res Ther (2012) 14(2):R66. doi: 10.1186/ar3782 22417776 PMC3446434

[33] Iaccarino L Ghirardello A Zen M Villalta D Tincani A Punzi L. Polarization of TH2 response is decreased during pregnancy in systemic lupus erythematosus. Reumatismo (2012) 64(5):314–20. doi: 10.4081/reumatismo.2012.314 23256107

[34] Yockey LJ Iwasaki A. Interferons and proinflammatory cytokines in pregnancy and fetal development. Immunity (2018) 49(3):397–412. doi: 10.1016/j.immuni.2018.07.017 30231982 PMC6152841

[35] Vilotić A Nacka-Aleksić M Pirković A Bojić-Trbojević Ž Dekanski D Jovanović Krivokuća M. IL-6 and IL-8: An overview of their roles in healthy and pathological pregnancies. Int J Mol Sci (2022) 23(23):14574. doi: 10.3390/ijms232314574 36498901 PMC9738067

[36] Nayak M Peinhaupt M Heinemann A Eekhoff MEW van Mechelen W Desoye G. Sedentary behavior in obese pregnant women is associated with inflammatory markers and lipid profile but not with glucose metabolism. Cytokine (2016) 88:91–8. doi: 10.1016/j.cyto.2016.08.031 27591509

[37] Ross KM Miller G Culhane J Grobman W Simhan HN Wadhwa PD. Patterns of peripheral cytokine expression during pregnancy in two cohorts and associations with inflammatory markers in cord blood. Am J Reprod Immunol (2016) 76(5):406–14. doi: 10.1111/aji.12563 27615067

[38] Witkin SS Gerber S Ledger WJ. Influence of interleukin-1 receptor antagonist gene polymorphism on disease. Clin Infect Diseases (2002) 34(2):204–9. doi: 10.1086/338261 11740709

[39] Steinkasserer A Spurr NK Cox S Jeggo P Sim RB. The human IL-1 receptor antagonist gene (IL1RN) maps to chromosome 2q14-q21, in the region of the IL-1 alpha and IL-1 beta loci. Genomics (1992) 13(3):654–7. doi: 10.1016/0888-7543(92)90137-H 1386337

[40] Simón C Valbuena D Krüssel J Bernal A Murphy CR Shaw T. Interleukin-1 receptor antagonist prevents embryonic implantation by a direct effect on the endometrial epithelium. Fertil Steril (1998) 70(5):896–906. doi: 10.1016/S0015-0282(98)00275-1 9806573

[41] Kimya Y Akdiş C Cengiz C Ozan H Tatlikazan S Uncu G. Plasma interleukin-1alpha, interleukin-1beta and interleukin-1 receptor antagonist levels in pre-eclampsia. Eur J Obstet Gynecol Reprod Biol (1997) 73(1):17–21. doi: 10.1016/S0301-2115(97)02698-5 9175684

[42] Bowen JM Chamley L Mitchell MD Keelan JA. Cytokines of the placenta and extra-placental membranes: biosynthesis, secretion and roles in establishment of pregnancy in women. Placenta (2002) 23(4):239–56. doi: 10.1053/plac.2001.0781 11969335

[43] Numasaki M Lotze MT Sasaki H. Interleukin-17 augments tumor necrosis factor-alpha-induced elaboration of proangiogenic factors from fibroblasts. Immunol Lett (2004) 93(1):39–43. doi: 10.1016/j.imlet.2004.01.014 15134897

[44] Numasaki M Takahashi H Tomioka Y Sasaki H. Regulatory roles of IL-17 and IL-17F in G-CSF production by lung microvascular endothelial cells stimulated with IL-1beta and/or TNF-alpha. Immunol Lett (2004) 95(1):97–104. doi: 10.1016/j.imlet.2004.06.010 15325804

[45] Numasaki M. Interleukin-17 promotes angiogenesis and tumor growth. Blood (2003) 101(7):2620–7. doi: 10.1182/blood-2002-05-1461 12411307

[46] Takahashi H Numasaki M Lotze MT Sasaki H. Interleukin-17 enhances bFGF-, HGF- and VEGF-induced growth of vascular endothelial cells. Immunol Lett (2005) 98(2):189–93. doi: 10.1016/j.imlet.2004.11.012 15860217

[47] Numasaki M Watanabe M Suzuki T Takahashi H Nakamura A McAllister F. IL-17 enhances the net angiogenic activity and *In vivo* growth of human non-small cell lung cancer in SCID mice through promoting CXCR-2-Dependent angiogenesis. J Immunol (2005) 175(9):6177–89. doi: 10.4049/jimmunol.175.9.6177 16237115

[48] Cornelius DC Lamarca B. TH17- and IL-17- mediated autoantibodies and placental oxidative stress play a role in the pathophysiology of pre-eclampsia. Minerva Ginecol (2014) 66(3):243–9. doi: 10.4049/jimmunol.175.9.6177 PMC5089699 24971780

[49] Pinheiro MB Martins-Filho OA Mota APL Alpoim PN Godoi LC Silveira ACO. Severe preeclampsia goes along with a cytokine network disturbance towards a systemic inflammatory state. Cytokine (2013) 62(1):165–73. doi: 10.1016/j.cyto.2013.02.027 23523008

[50] Liu H Liu A Kaminga AC McDonald J Wen SW Pan X. Chemokines in gestational diabetes mellitus. Front Immunol (2022) 13. doi: 10.3389/fimmu.2022.705852 PMC8860907 35211112

[51] de Freitas NPA Carvalho TR Gonçalves CCRA da Silva PHA de Melo Romão LG Kwak-Kim J. The dietary inflammatory index as a predictor of pregnancy outcomes: Systematic review and meta-analysis. J Reprod Immunol (2022) 152:103651. doi: 10.1016/j.jri.2022.103651 35696840

