# Supplementary material for: Panoramic snapshot of serum soluble mediator interplay in pregnant women with convalescent COVID-19: an exploratory study
Source: Front Immunol. 2023 Apr 12;14:1176898. doi: 10.3389/fimmu.2023.1176898 (PMC10130456; doi:10.3389/fimmu.2023.1176898)
Supplement: Supplementary file 1 [file DataSheet_1.pdf]

Supplementary Material

Panoramic snapshot of serum soluble mediator interplay in pregnant women with convalescent COVID-19: an exploratory study

Geraldo Magela Fernandes[1†*], Lizandra Moura Paravidine Sasaki[1,2†], Gabriela Profírio Jardim-Santos[1], Heidi Luise Schulte[1], Felipe Motta[1], Ângelo Pereira da Silva[2], Aleida Oliveira de Carvalho[2], Yacara Ribeiro Pereira[2], Caroline de Oliveira Alves[3], David Alves de Araújo Júnior[2], Dayde Lane Mendonça-Silva[2], Karina Nascimento Costa[3], Maria Eduarda Canellas de Castro[1,2], Lucas Lauand[3], Rodrigo de Resende Nery[3], Rosana Tristão[3], Patricia Shu Kurizky[1,2], Otávio de Toledo Nóbrega[1], Laila Salmen Espindola[1], Luiz Cláudio Gonçalves de Castro[1,3], Patrícia Nessralla Alpoim[4], Lara Carvalho Godoi[4], Luci Maria Sant Ana Dusse[4], Jordana Grazziela Alves Coelho-dos-Reis[5], Laurence Rodrigues do Amaral[6], Matheus de Souza Gomes[6], Pedro Luiz Lima Bertarini[6], Joaquim Pedro Brito-de-Sousa[7]; Ismael Artur da Costa-Rocha[7]; Ana Carolina Campi-Azevedo[7], Vanessa Peruhype-Magalhães[7], Andrea Teixeira-Carvalho[7], Alberto Moreno Zaconeta[3§], Alexandre Anderson de Sousa Munhoz Soares[1,3], Valéria Valim[8,9], Ciro Martins Gomes[1,3,10], Cleandro Pires de Albuquerque[1,2], Olindo Assis Martins-Filho[7§*], Licia Maria Henrique da Mota[1,2,10§]

*** Correspondence:** Geraldo Magela Fernandes & Olindo Assis Martins Filho. Universidade de Brasília Campus Universitário Darcy Ribeiro, Brasília, DF, 70910-900. Instituto René Rachou, Fundação Oswaldo Cruz. Avenida Augusto de Lima, 1715, Barro Preto, Belo Horizonte, MG, Brazil, 30190-002

1 Supplementary Figures

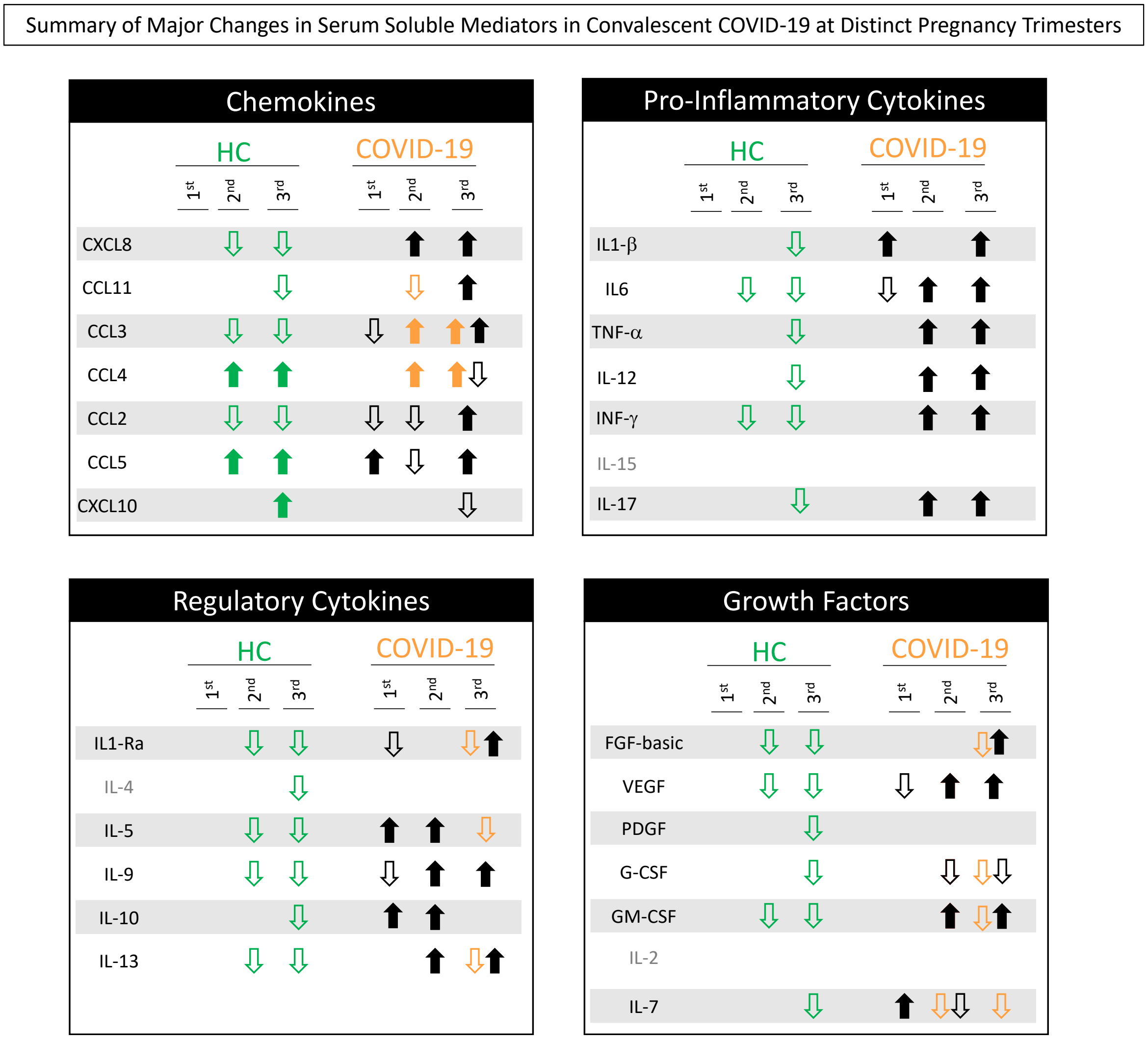

Supplementary Figure 1. *Summary of major changes in serum soluble mediators in convalescent COVID-19 at distinct pregnancy trimesters.* The levels of chemokines (CXCL8, CCL11, CCL3, CCL4, CCL2, CCL5, CXCL10), pro-inflammatory cytokines (IL-1β, IL-6, TNF-α, IL-12, IFN-γ, IL-15, IL -17), regulatory cytokines (IL-1Ra, IL- 4, IL-5, IL-9, IL-10, IL-13) and growth factors (FGF-basic, PDGF, VEGF, G-CSF, GM- CSF, IL-2, IL-7) were measured in serum samples from pregnant women with convalescent COVID-19 at 3-20 weeks after symptoms onset (COVID, n=89) and pre-pandemic non-infected pregnant women as a Healthy Control (HC, n=52). The HC and COVID-19 groups were further categorized into subgroups according to pregnancy trimester, referred as: HC 1st (n=21), HC 2nd (n=10), HC 3rd (n=21) and COVID 1st (n=7), COVID 2nd (n=34), COVID 3rd (n=48). The measurements were carried out by high-throughput multiplex bead array as described in Material and Methods. Multiple comparative analysis was performed by Kruskal-Wallis followed by Dunn's port-test and comparisons between COVID-19 and HC at matching pregnancy trimesters was assessed by Mann-Whitney test. In all cases, significance was considered at $p<0.05$. Intragroup significant differences were underscored by green and orange arrows, indicating decrease (⇩;⇩) or increase (⬆; ⬆) for comparisons between 2nd *vs* 1st and 3rd *vs* 1st trimesters. Inter-group significant differences at matching pregnancy trimesters were identified by black arrows, indicating decrease (⇩) or increase (⬆) for comparisons at 1st, 2nd and 3rd trimesters.

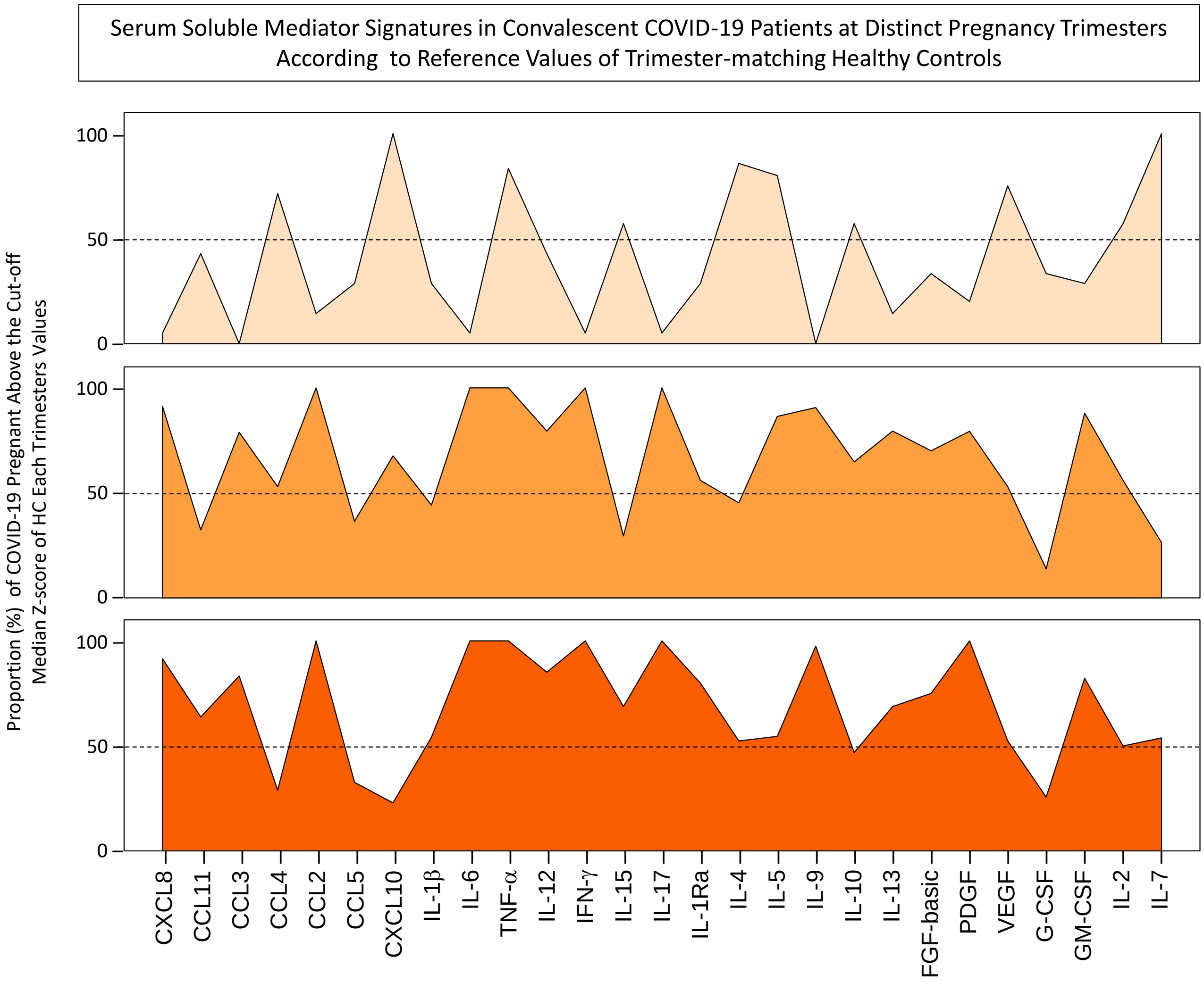

Supplementary Figure 2. *Serum soluble mediator signatures in convalescent COVID-19 patients at distinct pregnancy trimesters, according to reference values of trimester-matching healthy controls.* Signatures of: serum chemokines (CXCL8, CCL11, CCL3, CCL4, CCL2, CCL5, and CXCL10), pro-inflammatory cytokines (IL-1β, IL-6, TNF-α, IL-12, IFN-γ, IL-15, and IL-17), regulatory cytokines (IL-1Ra, IL-4, IL-5, IL-9, IL-10, and IL-13), and growth factors (FGF-basic, PDGF, VEGF, G-CSF, GM-CSF, IL-2, and IL-7) were assembled for pregnant women with convalescent COVID-19 at 3-20 weeks after symptom onset (COVID, n=89), categorized into subgroups according to pregnancy trimester, referred to as: COVID 1st (▭, n=7), COVID 2nd (▭, n=34), COVID 3rd (▭, n=48). The measurements were taken by high-throughput multiplex bead array as described in Material and Methods. The results are presented in line charts, underscoring the area under the curve, showing the proportion (%) of pregnant women with serum levels above the reference values (cut-off) defined as the median Z-score of each soluble mediator detected for HC subgroups at 1st, 2nd and 3rd trimesters as described in material and methods. The serum soluble mediators displaying a proportion of pregnant women above 50% (dashed line) were included in the set of biomarkers with increased levels.
